# Comparative long-term outcomes of vitrectomy combined with anterior chamber intraocular lens to intra-scleral haptic fixation of posterior chamber intraocular lens

**DOI:** 10.1186/s40942-024-00572-2

**Published:** 2024-08-26

**Authors:** Alexis Warren, Pavlina S. Kemp, Razek G. Coussa, Liang Cheng, H. Culver Boldt, Stephen R. Russell, A. Tim Johnson, Thomas A. Oetting, Elliott H. Sohn

**Affiliations:** Department of Ophthalmology and Visual Sciences, University of Iowa Healthcare, Iowa City, IA USA

**Keywords:** Vitrectomy, ACIOL, Yamane, Sutured IOL, Dislocated IOL, IOL exchange, Agarwal technique

## Abstract

**Purpose:**

To evaluate the long-term clinical outcomes in patients with combined pars plana vitrectomy (PPV) with anterior chamber intraocular lens (ACIOL) to intrascleral haptic fixation (ISHF) using the Agarwal technique with fibrin glue to secure the scleral flap of a posterior chamber intraocular lens.

**Methods:**

Retrospective, consecutive, single-center, comparative case series. 83 eyes were studied. Patients with < 8 months of follow-up were excluded. Detailed pre-, intra-, and post-operative complications were analyzed using mixed model univariate analysis and t-test. Pre- and post-operative best corrected visual acuity (BCVA) was analyzed.

**Results:**

Twenty-five subjects met entry criteria. Mean age at time of surgery was 70.4 ± 17.7 years in the ACIOL group (*n* = 12) and 54.6 ± 21.1 years in the ISHF group (*n* = 13; *p* = 0.03). Mean follow-up was 38.2 months. Incidence of corneal decompensation was similar in the ACIOL and ISHF lens group (*p* = 0.93). There was no difference in the BCVA mean change or cystoid macular edema (CME) at the final visit between the groups (*p* = 0.47; *p* = 0.08), but there was a trend toward increased CME in the ACIOL group.

**Conclusions:**

PPV with concomitant placement of either ACIOL or ISHF lens result in improvement in BCVA. Both procedures are well tolerated and result in favorable outcomes with long-term follow-up though varying patient populations do not allow precise comparison between the two groups.

**Supplementary Information:**

The online version contains supplementary material available at 10.1186/s40942-024-00572-2.

## Key messages

***What is known:***

•Posteriorly dislocated intraocular lenses (IOLs) can be challenging to treat and typically require vitrectomy and lens removal

•There is limited data comparing the long-term outcomes of exchange with different techniques in the setting of vitrectomy

***What is new:***

•Vitrectomy with concomitant ACIOL or intra-scleral haptic fixation may result in similar long term visual acuity gains

•Complication profile between the two groups are similar, with a trend toward increased cystoid macular edema in the ACIOL group

**Supplementary Information:**

The online version contains supplementary material available at 10.1186/s40942-024-00572-2.

## Introduction

Cataracts are the leading cause of blindness worldwide and their removal is the most commonly performed surgery in the United States Medicare population [1, 2]. However, in many cases, placement of an intraocular lens (IOL) inside the capsular bag is not feasible as in cases of trauma, poor capsular support or lens dislocation (aphakia) [3]. When inadequate capsular or structural support exists, the surgeon can choose amongst a number of options for alternatively fixated lenses that are currently available including anterior chamber (AC), iris fixation, and scleral-fixated intraocular lenses (IOLs) [4]. Despite the growing body of studies, no single secondary lens or technique has been proven to be superior to another [4–6]. Although most of these options are relatively safe procedures, they are not without potential post-surgical complications [3, 7]. To reduce these complications, placement of these IOLs may require concomitant pars plana vitrectomy (PPV), most commonly when there is a need to explant a dislocated IOL and/or remove retained lens fragments from the posterior segment.

Previous studies suggest that despite having an acceptable visual outcome, an ACIOL has a relatively higher rate of late complications, including uveitis-glaucoma-hyphema syndrome and cystoid macular edema (CME) [8], compared to posterior chamber IOLs [9–12]. Most notably, ACIOLs have been associated with endothelial cell loss and subsequent corneal decompensation [13, 14]. The exact mechanism for the endothelial cell loss remains unknown, as this is likely multifactorial [13–15]. Previous studies have highlighted direct mechanical trauma from the lens and/or subclinical inflammatory mediators for the morphologic changes and accelerated endothelial cell loss that is reported after successful ACIOL placement [16]. Other authors have noted that an association may be complicated by other confounding variables including intraoperative surgical microtrauma from surgical instrumentation which may be present regardless of lens type [15]. 

Innovative suture and scleral-fixation techniques have been developed to improve visual acuity outcomes or expand the margin of error for procedure completion while decreasing the complication rate associated with secondary IOL placement [4, 5, 17]. As these alternate lens implantation techniques arise, studies are needed to detail the differences in the long term visual outcomes between ACIOL and scleral-fixated posterior chamber IOL [9, 18, 19] with modified Agarwal technique as no other study has evaluated these two options together specifically in the setting of concurrent PPV, which is required in instances like IOL dislocation to the posterior segment. The goal of our study was to evaluate the long-term clinical outcomes in patients with PPV combined with placement of anterior chamber intraocular lens (ACIOL) and posterior chamber intra-scleral-fixated fibrin-glue-assisted lenses (ISFH).

## Methods

This was a retrospective, consecutive, comparative case series of patients who underwent PPV with concomitant placement of either ACIOL or ISHF at the University of Iowa Hospitals and Clinics from 2000 to 2018. All experimental protocols were approved by the Institutional Review Board (IRB) committee at the University of Iowa, who waived the need for informed consent. All methods were carried out in accordance with relevant guidelines and regulations, including the Declaration of Helsinki. Potential subjects were identified through personal physician case log and common procedure terminology (CPT) codes such as 996.53 (mechanical complication due to intraocular lens) and 379.34 (posterior dislocation of lens). Baseline subject characteristics were documented including age, sex, pre-existing ocular conditions and etiology for capsular and zonular insufficiency.

### Subject selection

Indications for secondary lens surgery included traumatic cataract with lens subluxation, subluxed intraocular or crystalline lens, capsular tear/rupture, IOL exchange, aphakia (primary loss or prior surgery), and phacodonesis. These cases were reviewed to confirm inclusion of those that received combined PPV to address posterior pathology such as lens retrieval or retinal detachment repair. Patients with less than 8 months post-operative follow-up were excluded to assure stable visual outcomes. Intraocular lens selection was determined by surgeon preference and some patient factors such as reasons for previous lens explantation, patient age, and other ocular anatomic considerations. Subject charts were reviewed to document pre-, intra- and-post-operative data including suprachoroidal hemorrhage, choroidal detachment, lens subluxation, uveitis-glaucoma-hyphema syndrome, corneal decompensation, hyphema, glaucoma, cystoid macular edema, vitreous hemorrhage, epiretinal membrane and retinal detachment. Snellen best corrected visual acuity (VA) was documented both at the pre-operative and final post-operative visit and converted to the logarithmic minimum angle of resolution (logMAR) equivalent.

### Surgical techniques

The surgical technique included a standard 23- or 25-gauge three port PPV with a phacofragmatome in cases of sclerotic retained lens material that could not be removed with the cutter. Posteriorly dislocated IOLs were lifted using the extrusion needle into the anterior vitreous where a 23G low mass pick-forcep could securely grasp the IOL to occasionally place it on the iris where viscoelastic device was already placed in the anterior chamber for protection. More often, the clear corneal or scleral tunnel wound described below would be created at the time the IOL was being elevated so it could be removed without resting on the iris. Before placement of the new IOL scleral depression was performed for 360-degrees and any retinal tears were lasered.

Anterior chamber lens implantation was performed by the vitreoretinal surgeon or anterior segment surgeon: a caliper was used to measure the horizontal white-to-white dimension. A superior peritomy was performed and a 6 mm scleral groove was made into the clear cornea. If necessary, PPV was performed and the lens complex (if applicable) was brought into the anterior chamber and secured with a Duet forceps (MicroSurgical Technology [MST], Redmond, WA). A 2.75 mm keratome blade (BVI, Waltham, MA) was then used to enter the anterior chamber forming a temporal corneal tunnel. The lens/capsule complex was partially bisected and then prolapsed through the incision for explantation [20]. An Alcon MTA3-5UO (Alcon Laboratories, Fort Worth, TX) intraocular lens was inserted into through the corneal incision, guiding the leading haptic into the nasal iridocorneal angle, and trailing haptic was directed to rest easily in the sub-incisional angle. An iridectomy was performed. The wound was closed with 10 − 0 nylon suture and sclerotomies closed with 6 − 0 plain gut suture.

The surgical technique for placement of the ISHF with fibrin glue, first described by Agarwal, [21] is illustrated in the Supplementary video and detailed here: a toric marker is used to indicate the location of the haptics on the cornea. A peritomy is performed before a 300-micron steel blade is used to fashion a 2.5 mm square flap, and a crescent blade used to dissect the flaps into clear cornea. Vitrectomy was performed by the vitreoretinal surgeon and any posterior segment pathology was addressed. If dislocated posteriorly, the IOL was brought into the anterior chamber and explanted through the scleral-corneal wound. The eye was entered under the flaps approximately 1.5 mm from the limbus using the side port blade. A keratome blade was used to enter the anterior chamber forming a corneal tunnel. An Alcon MA50BM lens was inserted into the eye, leaving the trailing haptic protruding through the corneal wound outside the anterior chamber. The Duet micrograspers were used to grasp and externalize the tip of the haptic through the scleral opening. The trailing haptic was brought into the eye and externalized through the other scleral opening using a handshake technique. Scleral tunnels for tucking the haptic were made with a 26-gauge needle. Each of the haptics were tucked into the pockets and adjusted to achieve centration of the lens. If there was significant tilt despite haptic tuck adjustment, a new scleral entry point was made. The vitreoretinal surgeon checked the retinal periphery for tears with careful depression around the scleral tunnels to ensure the haptics were not moved resulting in lens decentration. An air bubble was placed in the anterior chamber. The scleral flaps and overlying conjunctiva were secured using Tisseel© fibrin glue (Baxter, Deerfield, IL).

### Statistical analysis

Visual acuity (VA) analysis was performed on patients with at least 8 months of follow up and was converted to logMAR units for standardization. For patients presenting with limited vision such as counting fingers, the acuity was documented as 1/200 or a logMAR equivalent of 2.3. For hand motions, light perception, and no light perception logMAR VA of 3.3, 4.3 and 5.3 were assigned, respectively. A mixed model univariate analysis was then carried on the logMAR pre- and post-operative change in BCVA with respect to type of intraocular lens and number of pre-operative visually significant comorbidities listed above and were intra and post-operative complications. T-tests were used for comparative analysis between the two studied groups. Microsoft Excel and IBM SPSS Statistics (version 26) were used for the statistical analysis. Statistical significance was set at *p <* 0.05 for all comparisons.

## Results

Eighty-three patients were identified through personal physician case logs and searched CPT codes for secondary lens surgery. Patients who did not have combined PPV (28 patients) or less then 8 months of follow up (28 patients) were excluded; two patients had a sulcus IOL placed that resulted in exclusion (Supplementary figure). Twenty-five eyes from 25 patients met the inclusion and exclusion criteria and were analyzed that were operated on by six surgeons. Baseline demographics are detailed in Table 1. Twelve patients underwent combined PPV with implantation of ACIOL (five female patients) and 13 patients underwent combined PPV with implantation of ISHF fibrin glued lenses (two female patients). Mean age at presentation was 62.2 years (median 67, range 19–92). Mean age in the ACIOL group was 70.4 years (median 74, range 27–92) while the mean age of the fibrin-glued group was 54.6 years (median 57, range 19–83; *p* = 0.027). The mean time to follow-up was 38.2 months (median 27.6 months, range 8-215 months).

**Table 1:** Demographics and pre-operative ophthalmic characteristics of the anterior chamber intraocular lens (ACIOL) and intra-scleral haptic fixation (ISFH) with glued flap lens groups

	ACIOL	ISHF	*p* value
Number of patients	12	13	
Mean age (range)	70.4 (27–92)	54.6 (19–83)	0.03*
Sex	5 Females	2 Females	
Mean follow-up (months)	44.3	32.7	0.25
Mean preop VA (logMAR)	1.469	0.8262	0.07
**Surgical Indications**			
Traumatic cataract with subluxation	0	1	
Subluxed intraocular lens	7	5	
Subluxed crystalline lens	0	2	
Capsular tear/rupture	3	1	
IOL exchange	0	1	
Aphakia	1	3	
Phacodonesis of crystalline lens	1	0	
**Pre-operative co-morbidities**			0.35
Glaucoma	3	3	
Myopia	1	1	
Uveitis	1	1	
Diabetes	0	2	
AMD	3	0	
ERM	2	1	
CME	1	0	
Retinal detachment	6	6	

Abbreviations IOL: intraocular lens; ACIOL: anterior chamber intraocular lens; ISHF: intrascleral haptic fixated; VA: visual acuity; CME: cystoid macular edema; ERM: epiretinal membrane; AMD: age-related macular degeneration

Pre-operative indication for surgery included aphakia, dislocated IOL, subluxed crystalline lens for which anterior approach would have a reasonably high chance of complete dislocation, and previous significant capsular loss precluding use of a sulcus IOL. The mean number of pre-operative visually significant diagnoses was 1.25 in the ACIOL group and 1.08 in the ISHF lens group (*p* = 0.47). Eleven of the 12 patients (91.6%) in the ACIOL group had undergone at least one previous surgery in the enrolled eye compared to ten of the 13 patients (76.9%) in the ISHF group.

The mean pre-operative Snellen equivalent VA across all eyes was logMAR 1.14 (20/273). In the ACIOL group, the mean presenting logMAR VA improved from 1.47 (20/587) to 1.23 (20/341) at the final visit (*p* = 0.35). In the ISHF lens group, the mean presenting logMAR VA improved from 0.83 logMAR (20/134) to 0.65 (20/90) at the final visit (*p* = 0.3). The mean overall logMAR change in VA for both groups were − 0.205 with no statistical difference between the groups (-0.24 and − 0.18, respectively *p* = 0.47).

Complication profiles, both intraoperative and post-operative, were similar between the two lens groups. As summarized in Table 2, intra-operatively there was no suprachoroidal hemorrhage or choroidal detachment in either the ACIOL or ISHF lens group. Ten of 12 eyes (83.3%) in the ACIOL group had at least one post-operative complication compared to eight of 13 eyes (61.5%) in the ISHF lens group. There was no difference in the number of post-operative complications between the ACIOL and ISHF lens group (1.67 vs. 1.08, *p* = 0.097). The most common complication in the cohort was corneal decompensation which was present in 32% of the studied eyes (*n* = 8) followed by CME at 28% (*n* = 7). Other post-operative complications in the overall cohort included suprachoroidal hemorrhage in 8% of eyes (*n* = 2), lens subluxation in 16% of eyes (*n* = 4), epiretinal membrane in 12% (*n* = 3), and vitreous hemorrhage in 16% (*n* = 4) (Table 2). There were no post-operative retinal detachments, glaucoma or uveitis-glaucoma-hyphema (UGH) syndrome in either group. There were no differences in the incidence of post-operative complications between the ACIOL and ISHF lens groups (Table 2). Six eyes, in both the ACIOL (25%, *n* = 3) and ISHF (23%, *n* = 3) lens group ultimately required keratoplasty. 16.7% of eyes (*n* = 2) in the ACIOL group required repeat keratoplasty at three and eight years post-operatively. One eye in the ACIOL group (8.3%) underwent two penetrating keratoplasties, which was further complicated by perforation and ultimately enucleation.

**Table 2:** Peri-operative complications associated with anterior chamber intraocular lens and intra-scleral haptic fixation with fibrin glued flap lens groups

Complications		ACIOL	ISHF lens	*p*-value
Intra-operative	Suprachoroidal hemorrhage	0	0	n/a
	Choroidal detachment	0	0	n/a
Post-operative	Corneal decompensation	4	4	0.93
	Hyphema	0	0	n/a
	Unstable IOL	2	2	0.98
	UGH	0	0	n/a
	Glaucoma	0	0	n/a
	Retinal detachment	0	0	n/a
	CME	6	1	0.08
	ERM	2	1	0.73
	VH	2	2	0.98
	Suprachoroidal hemorrhage	2	0	0.50
	Choroidal detachment	0	0	n/a

Abbreviations IOL: intraocular lens; ACIOL: anterior change intraocular lens; ISHF: intrascleral haptic fixated; UGH: uveitis-glaucoma-hyphema syndrome; CME: cystoid macular edema; ERM: epiretinal membrane; VH = vitreous hemorrhage

## Discussion

In this study, both ISHF fibrin-glued PCIOL and ACIOLs techniques with vitrectomy were well-tolerated and resulted in VA improvement as has been previously reported (albeit with shorter duration of follow-up) [22]. We utilized a multi-specialty, team-based approach to compare two lens implantation techniques, as little direct comparative data exists looking at the long-term complication rates for these primarily in the setting of concurrent PPV. Although there has been some comparative data to suggest ACIOLs and ISHF lenses are equally efficacious [18], to our knowledge, there has not been a long-term follow-up study comparing outcomes of ISHF fibrin-glued PCIOLs to ACIOLs with combined vitrectomy surgery.

It is important to note that both groups had similar presenting pre-operative VA that were similar to the studies previously reported [23]. Our post-operative and end-of-follow-up visual outcomes were similar in both groups. This contrasts to the visual outcomes reported by Omlecki et al [24] who showed a statistically significant improved visual acuity with the use of an ACIOL compared to the scleral-fixated PCIOL. These differences may be accounted for by the differences in mean follow-up time between our study and theirs (38.2 months versus 4.8 months respectively), which may imply that visually significant complications from ACIOLs may manifest in the late post-operative period. Post-operative complications in our study were generally similar between the two lens groups, with no significant difference in the rate of corneal decompensation and retinal complications.

We found a non-significant trend towards more post-operative CME in the ACIOL group compared to the ISHF IOL (Table 2). Increased CME in ACIOLs has not been documented previously although this can occur after any IOL placement [4, 5]. Finn et al. described 50 eyes undergoing ACIOL implantation with concurrent PPV with a lower rate of CME at 15% compared to our ACIOL study group rate of 50%. Interestingly, although still a minority of patients, the post-operative CME complication rate has previously been documented at an increased rate compared to their equivalent SFIOLs, but the significance remains unclear especially given that CME appears to be one of the more common post-operative complications regardless of lens type [23, 25].

Use of an ACIOL in cases of poor capsular support remains a topic of debate due to its associated complications including endothelial cell death and CME [7] increasing the importance of alternate PCIOL techniques [26]. However, a PCIOL sutured at the ciliary sulcus has been associated with complications such as vitreous hemorrhage, retinal detachment and lens destabilization [5, 27]. Fibrin-glue has been used for several off-label indications in ophthalmology including sealing of post cataract corneal wounds and securing of conjunctival grafts with adequate success [28, 29]. Traditionally, the fibrin glue has been highlighted for its ability to provide good hemostasis and offer enhanced rate of adhesion with reduced surgical time and as such has been more recently employed as a technique for suture-less intraocular lens implants [21]. This was first described by Agarwal et al. [21] in 2008 and has since undergone modifications to become the technique described in this study. Thus far, the post-operative complications from this technique appear to be equal if not less than their anterior chamber counterparts, especially in those with aphakia [8, 30]. Other suture-related complications such as suture erosion, exposure and disintegration are also avoided using the glued technique which may account for the reduced infection rate as the fixated haptics are well covered by the sclera [30, 31]. Based on these findings in addition to their reduced operative time, the glued IOL may be a preferred technique for combined procedures, especially in the setting of poor zonular support [30]. 

Little attention has been drawn to concurrent removal of vitreous during scleral-fixated PCIOLs which may further decrease complication rates. Many anterior segment surgeons performing secondary lens surgery are most comfortable performing limbal based anterior vitrectomy, which allows for faster operating time. In contrast, Nabors et al [32] suggested that complete PPV with concurrent insertion of ciliary sulcus PCIOL may decrease retinal complications. Tsunoda and colleagues [33] confirmed in 30 pig eyes that anterior vitrectomy alone was not enough to remove all the vitreous and the capsule remains at the ciliary sulcus. Thus, one could expect increased vitreous capture of the PCIOL haptics causing an increase in post-operative CME and retinal detachment [27, 33]. Complete PPV might reduce vitreous traction resulting in lower incidence of post-operative retinal complications that we observed.

There are a few limitations to this study aside from its retrospective nature and limited number of patients. The mean age of the ACIOL patients in were older than the ISFH group which may account for subtle differences in the post-operative complications likely due to pre-surgical ocular comorbidities and endothelial health; mitigation was attempted with use of mixed models statistical analysis. The tendency to use an ACIOL instead of sutured or fixated PCIOL in older patients reflect the arguable notions that (1) suture materials have a limited life, (2) surgical time for an ACIOL insertion is shorter making ISFH IOL less appealing to perform in those who may not tolerate a longer procedure (e.g. elderly patients and those with multiple co-morbidities), and/or (3) late complications may be seen more frequently in those with ACIOLs [9]. A prospective study comparing ACIOL and ISFH IOLs with similar enrollment for pre-operative characteristics including age would be valuable to determine whether the short and long-term outcomes between these groups are truly different. In addition, our series included patients with significant ocular comorbidities such as uveitis and retinal detachment of which half were macula off. This would have affected the pre- and post-operative visual acuities which could make this study less applicable to more routine secondary lens surgeries. The postoperative astigmatism from larger ACIOL incisions may also play a role in refractive outcomes that was not accounted for due to lack of complete, final refractive data. Moreover, despite long term follow-up, the smaller sample size may account for the lack of statistical significance between the VA outcomes of the two lens group and ultimately limits some of its clinical application.

We recommend that secondary lens choice be guided by surgeon comfort, expertise and patient-specific factors such as baseline anatomy. We suggest the use of an ACIOL where a large incision is already indicated for explantation of a dislocated polymethyl methacrylate (PMMA) IOL or dense Soemmerings ring that cannot be removed with the vitrector. More aggressive use of anti-inflammatory drops should be considered in the case of ACIOLs for CME prophylaxis. Despite corneal decompensation being equally likely in our series, many cornea surgeons may prefer posterior chamber IOLs for combined keratoplasty procedures.

### Electronic supplementary material

Below is the link to the electronic supplementary material.

Supplementary Material 1

Supplementary Material 2

## Data Availability

No datasets were generated or analysed during the current study.
