# Supplementary material for: Comparative long-term outcomes of vitrectomy combined with anterior chamber intraocular lens to intra-scleral haptic fixation of posterior chamber intraocular lens
Source: Int J Retina Vitreous. 2024 Aug 26;10:59. doi: 10.1186/s40942-024-00572-2 (PMC11346030; doi:10.1186/s40942-024-00572-2)
Supplement: Supplementary file 2 — Supplementary Material 2 [file 40942_2024_572_MOESM2_ESM.pptx]

## Slide 1
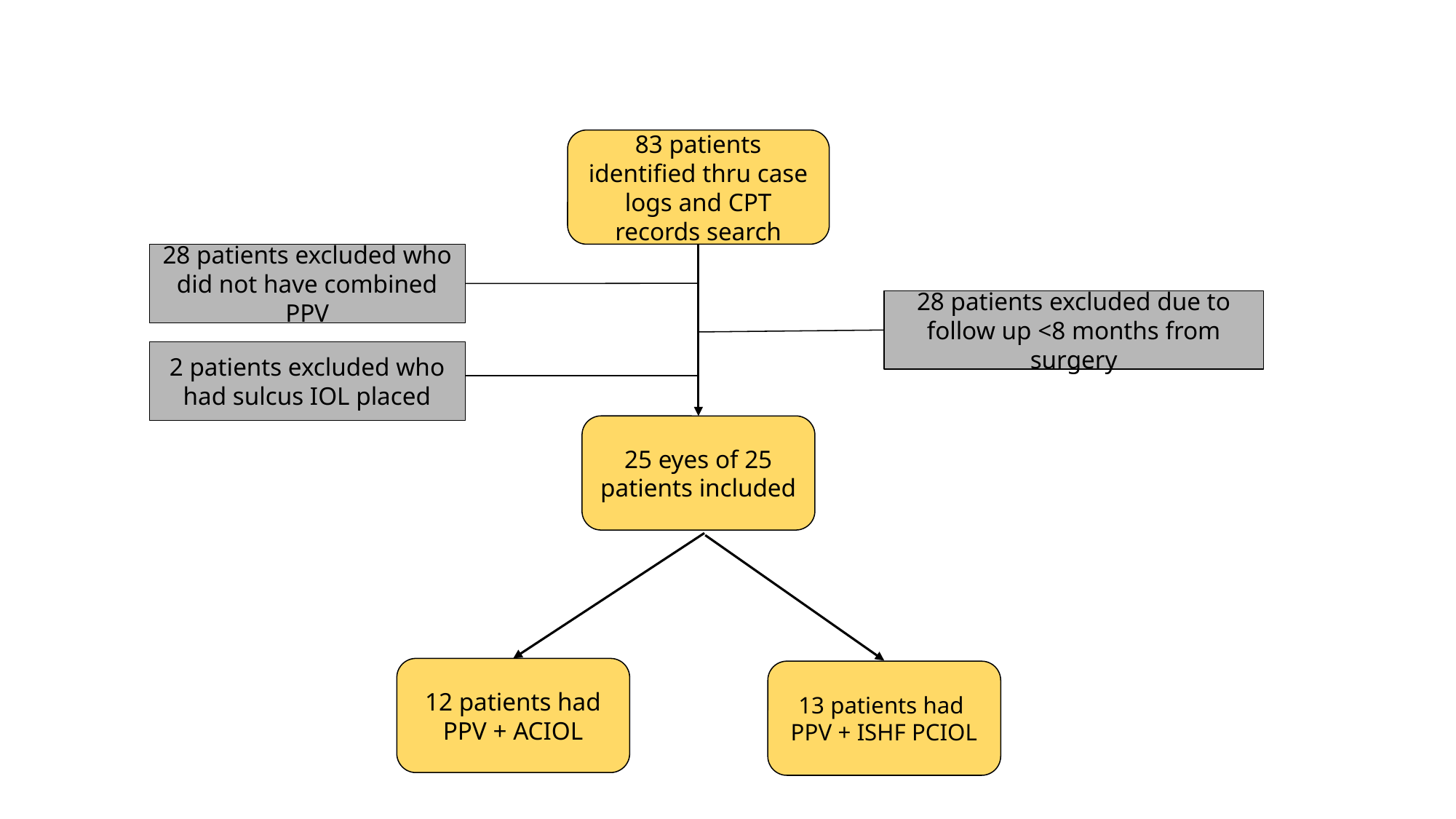

83 patients identified thru case logs and CPT records search
28 patients excluded who did not have combined PPV
28 patients excluded due to follow up <8 months from surgery
2 patients excluded who had sulcus IOL placed
25 eyes of 25 patients included
12 patients had PPV + ACIOL
13 patients had
PPV + ISHF PCIOL
